# A novel *Lactiplantibacillus plantarum* strain: probiotic properties and optimization of the growth conditions by response surface methodology

**DOI:** 10.1007/s11274-023-03862-3

**Published:** 2024-01-09

**Authors:** Gökhan Gurur Gökmen, Seda Sarıyıldız, Remzi Cholakov, Ayşe Nalbantsoy, Biray Baler, Emek Aslan, Ahmet Düzel, Sait Sargın, Yekta Göksungur, Duygu Kışla

**Affiliations:** 1 Engineering Faculty, Food Engineering Department, Ege University, Bornova, Izmir, Türkiye; 2 Kaasmakerij Özgazi, Nijverheidsweg 39, 4879 AP Etten-Leur, The Netherlands; 3 Engineering Faculty, Bioengineering Department, Ege University, Bornova, Izmir, Türkiye; 4 Agricultural Faculty, Agricultural Biotechnology Department, Ondokuz Mayıs University, Atakum, Samsun, Türkiye; 5 Faculty of Engineering and Architecture, Bioengineering Department, Sinop University, Nasuhbasoglu, Sinop, Türkiye; 6 Faculty of Engineering and Natural Sciences, Department of Bioengineering, Bursa Technical University, Yildirim, Bursa, Türkiye

**Keywords:** Probiotic, *Lactiplantibacillus plantarum*, Biomass production, Response surface methodology

## Abstract

The objective of this study is to explore the probiotic properties and optimal growth conditions of *Lactiplantibacillus plantarum* BG24. *L. plantarum* BG24 exhibited a remarkable ability to utilize lactose, and to grow under acidic conditions and in the presence of high levels of bile salts. The strain showed the highest antibacterial activity against *L. monocytogenes* Scott A (zone of inhibition: 26 mm). *L. plantarum* BG24 was found to be resistant to 8 of the tested 19 antibiotics using the disc diffusion method.and its multiple antibiotic resistance (MAR) index was calculated as 0.421. The adhesion rate to human intestinal epithelial Caco-2 cells was determined as 37.51%. The enzyme profile of *L. plantarum* BG24 was investigated using API ZYM test kit and the highest enzymatic activities were found for Leucine arylamidase, β-glucosidase, Valine arylamidase, β-galactosidase and N-acetyl-β-glucosaminidase. *L. plantarum* BG24 strain showed higher microbial growth under static conditions (6.60 OD_600_) compared to 100 rpm (5.73 OD_600_) and 200 rpm (5.02 OD_600_) shaking speed due to its facultative anaerobic characteristic. However, different inoculation rates and glucose addition did not make a statistically significant difference on biomass formation (p > 0.05). The specific growth rate of *L. plantarum* BG24 was 0.416 h^−1^, the doubling time was 1.67 h, and the biomass productivity value was 0.14 gL^−1^ h^−1^ in the original MRS broth (pH 5.7) while higher values were found as 0.483 h^−1^, 1.43 h and 0.17 gL^−1^ h^−1^, respectively, in MRS broth (pH 6.5) medium enriched with 5 g/L yeast extract. The stirred tank bioreactor was used to optimise the growth of BG24 strain. The process variables was optimized at 0.05 vvm of aeration rate, 479 rpm of agitation speed, 3% of inoculation rate and 18 h of incubation time. The maximum biomass (g/L) production was obtained as 3.84 g/L at the optimized conditions.

## Introduction

Boza which is a traditional fermented beverage is produced by lactic acid bacteria (LAB), yeast and various cereals (especially cooked maize, millet, rice or wheat semolina/flour). Thanks to the protein, carbohydrate, fiber, fat and lactic acid contents of boza, it contributes human nutrition (Ilango and Antony 2021). Various LAB and yeast exist in boza beverage between 2.94 × 10^5^ and 4.6 × 10^8^ cfu/mL, between 2.24 × 10^5^ and 8.40 × 10^7^ cfu/mL, respectively (Altay et al. 2013). The probiotics are defined as “live microorganisms which confer a health benefit on the host, when administered in adequate amounts” (WHO/FAO 2002). The viable cell count level of a probiotic products can differ according to countries. In Japan, at least 10^7^ cfu/g of probiotic should be exist in a food sample to be consider as a probiotic, however in United States a product can be labeled as probiotic if only it contains a minimum of 10^8^ cfu/g probiotic bacteria (Fatima Sherwani and Ara Abbas Bukhari 2022). The major genera used in probiotic products are grouped as (i) *Bifidobacterium, Lactobacillus, Streptococcus* which are lactic acid producing; (ii) *Bacillus, Propionibacterium* which are non-lactic acid producing; (iii) *Saccharomyces* that is non-pathogenic yeasts; (iv) rod or coccobacilli group which are non-spore forming and non-flagellated (Saraf et al. 2010). According to the WHO/FAO (2002), probiotic microorganisms must accomplish some properties such as producible in industrial scale, survivability during production and storage, tolerance to the gastrointestinal (GI) tract, having beneficial health effects to the host. Furthermore, it is advisable for probiotic strains to exhibit industrially significant attributes, such as heat resistance, particularly in the context of spray drying processes, and the capacity for exopolysaccharide (EPS) synthesis. These features can present advantages to consumers, both in the form of non-digestible dietary fiber and the enhancement of food palatability.

*L. plantarum*, situated within the taxonomic framework of the genus *Lactobacillus*, is a member of the facultatively heterofermentative group of lactobacilli. This species demonstrates a heterogeneous and versatile character, manifesting its presence across an array of ecological habitats, encompassing dairy, meat, fish, as well as numerous vegetable or plant fermentations. Various *L. plantarum* strains have been already used as starter cultures to produce a wide range of fermented foods and beverages with a good taste and texture (Behera et al. 2018). *L. plantarum* boasts a substantial historical record of secure utilization in the preparation of fermented foods, and it holds a prominent status as a constituent of the GI tract microflora (Swain et al. 2014). An assortment of both ethnically rooted and commercially available probiotic formulations are presently accessible in the marketplace, all of which rely on the foundation of *L. plantarum* (Swain et al. 2014). Although probiotics have a lot of potential benefits, judicious discernment is requisite in the selection of a probiotic supplement, given that not all strains have substantiated efficacy. Furthermore, it is imperative to emphasize that probiotics should not be regarded as a surrogate for a healthful diet and lifestyle; instead, they should be considered as a supplementary adjunct to these foundational elements.

In this study, the potential probiotic properties of *L. plantarum* BG24 strain isolated from boza was investigated. For this purpose, the survivability of *L. plantarum* BG24 was determined at different pH values (2.0, 3.5 and 5.0) and bile salt concentrations (0.5, 1.0, 1.5 and 2.0%, w/v). Subsequently, the study examined the microbial growth of *L. plantarum* BG24 at various temperatures (10, 25, and 45 °C) and NaCl concentrations (4.0 and 6.5% w/v). Moreover, investigations were conducted to evaluate lactose utilization, antibacterial activity against both Gram-positive (*Staphylococcus aureus* ATCC 6538P, *Listeria monocytogenes* Scott A) and Gram-negative (*Escherichia coli* O157:H7 ATCC 35150, *Salmonella* Typhimurium NRRL-B4420) pathogens, adhesion to human intestinal epithelial Caco-2 cells, multiple antibiotic resistance (MAR) index, and enzyme profiles of *L. plantarum* BG24. In the second step, the optimal growth conditions of *L. plantarum* BG24 were ascertained by examining the influence of various parameters. These parameters included inoculum ratios (1, 2, 3, 4, and 5% v/v), pH levels of the growth medium (5.5, 5.7, 6.0, 6.3, 6.5, 6.7, and 7.0), agitation rates (0, 100, and 200 rpm), addition of glucose (5, 10, 15, and 20 g/L), and yeast extract (5 and 10 g/L) on microbial growth. Furthermore, a correlation between optical density and biomass was established. Finally, the growth of the bacteria in a 5-liter stirred tank bioreactor was optimized using response surface methodology (RSM).

## Materials and methods

*Lactiplantibacillus plantarum* BG24 (Accession number: OQ625293) was isolated from naturally fermented boza and identified by 16 S rRNA sequencing (ARDRA). *L. acidophilus* LA5 (Christian Hansen, Hoersholm, Denmark) and *L. plantarum* Visby (VISBYVAC®, Serie 1000- Product Nr. 40022951, Germany) were used as reference probiotic LAB strains in the study. The pathogen bacteria strains used in the study were “*Salmonella* Typhimurium NRRL-B4420, *Staphylococcus aureus* ATCC 6538P, *Escherichia coli* O157:H7 ATCC 35150, *Listeria monocytogenes* Scott A”. All LAB and pathogen bacteria strains were gently supplied by Food Microbiology Department, Ege University, Izmir, Türkiye and were stored at − 86 °C in 25% glycerol stocks until used. The LAB cultures were grown in de Man, Rogosa and Sharpe (MRS; pH 5.7 ± 0.2, Merck, Germany) broth and MRS agar (pH 5.7 ± 0.2; Merck, Germany) at 37 °C. MRS broth/agar medium is used routinely for the growth of various *Lactobacillus* strains since it is a both rich and expensive medium which supplies the complex growth requirements of Lactobacilli strains (Horn et al. 2005; Papizadeh et al. 2020). The pathogen bacteria were grown in Tryptic Soy Broth (TSB; pH 7.3 ± 0.2, Merck, Germany) and/ or Tryptic Soy Agar (TSA; pH 7.3 ± 0.2, Merck, Germany). Human colon colorectal adenocarcinoma (Caco-2, ATCC-HTB-37) cells were gently supplied by Bioengineering Department, Ege University, Izmir, Türkiye.

### Investigation of the probiotic potential of *L. plantarum* BG24

#### Tolerance to low pH and bile salt

Tolerance to low pH of *L. plantarum* BG24 and reference strains (*L. acidophilus* LA5 and *L. plantarum* Visby) was determined by using the method of Pundir et al. (2013). Briefly, active bacterial culture in MRS broth were inoculated (1.0%, v/v) in 10 mL of fresh MRS broth tubes adjusted to pH 2.0, 3.5 and 5.0 with hydrochloric acid (1.0 or 6.0 N) and incubated for 3 days at 37 °C. Next, 0.1 mL aliquots of each tube were inoculated to MRS agar by pour plating method and incubated for 48 h at 37 °C. The microbial growth observed on MRS agar was designated as pH tolerant. To examine the bile salt tolerance of *L. plantarum* BG24 and reference strains, the MRS broth with varying concentrations of bile salt (0.5, 1.0, 1.5 and 2.0%, w/v) was inoculated with each active LAB culture (1.0%, v/v) and incubated at 37 °C for 48 h. Then, 0.1 mL of sample was inoculated to MRS agar by pour plate method and incubated for 48 h at 37 °C. The microbial growth observed on MRS agar was designated as bile salt tolerant (Pundir et al. 2013).

#### Survival under different temperature and NaCl conditions

*L. plantarum* BG24 and reference strains were activated in MRS broth and inoculated (1%, v/v) in 10 mL of fresh MRS broth tubes and incubated at 10 °C, 25 °C and 45 °C for 48–72 h. After the incubation, 0.1 mL inoculum were transferred separately to MRS agar by pour plating method and incubated for 48 h at 37 °C. The microbial growth on MRS agar media was used to designate the isolates as temperature tolerant (Tambekar and Bhutada 2010). The active LAB cultures were also inoculated (1%, v/v) in 10 mL of MRS broth medium with the concentrations of 4.0 and 6.5% NaCl (w/v) and incubated for 3 days at 37 °C. After the incubation time, 0.1 mL inoculum from each tube were inoculated to MRS agar by pour plating technique and incubated for 48 h at 37 °C. The microbial growth observed on MRS agar was designated as NaCl tolerant (Pundir et al. 2013).

#### Lactose utilization

The lactose utilization of *L. plantarum* BG24 and reference strains was determined by using the method by (Pundir et al. 2013). Sterilized medium (15 g/L NaCl, 10 g/L peptone, 5 g/L lactose, 0.018 g/L phenol red; pH 7.0 ± 0.2) was inoculated with active LAB cultures (1%, v/v) and incubated for 48 h at 37 °C. The color change red to yellow was considered as the production of acid by LAB.

#### Antibiotic resistance

The antibiotic resistance of *L. plantarum* BG24 and reference strains was detected by using agar disc diffusion method by Pundir et al. (2013). The LAB strains were activated in MRS broth medium and adjusted to 0.5 Mcfarland standards (≅1.5 × 10^8^ cfu/mL) in sterile Phosphate Buffered Saline (PBS; pH 7.4). The bacterial cell suspension was diluted 10-fold and 0.1 mL aliquots were inoculated on MRS agar plates by spread plating technique. The antibiotic discs were aseptically placed on the surface of agar and then petri plates were incubated for 48 h at 37 °C. Susceptibility pattern was assessed using 19 different antibiotics which are Amikacin (30 µg/disc, Oxoid, England), Amoxicillin (25 µg/disc, Oxoid, England), Ampicillin (10 µg/disc, Oxoid, England), Cefamandole (30 µg/disc, Oxoid, England), Chloramphenicol (30 µg/disc, Oxoid, England), Ciprofloxacin (5 µg/disc, Oxoid, England), Doxycycline (30 µg/disc, Oxoid, England), Erythromycin (15 µg/disc, Oxoid, England), Gentamicin (10 µg/disc, Oxoid, England), Kanamycin (30 µg/disc, Oxoid, England), Lincomycin (15 µg/disc, Oxoid, England), Nalidixic acid (30 µg/disc, Oxoid, England), Penicillin (10 E/disc, Oxoid, England), Piperacillin (100 µg/disc, Oxoid, England), Oxacillin (1 µg/disc, Oxoid, England), Rifampin (5 µg/disc, Oxoid, England), Tetracycline (30 µg/disc, Oxoid, England), Tobramycin (10 µg/disc, Oxoid, England) and Vancomycin (30 µg/disc, Oxoid, England). The zone of inhibition diameters (ZOI) was measured and interpreted (Katiku et al. 2022) as ZOI ≥ 21 mm (Sensitive, S); ZOI: 16–20 mm (Intermediately susceptible, I); ZOI ≤ 15 mm (Resistant, R).

Multiple antibiotic resistance (MAR) of each bacterium were determined to use in the calculation of the probiotic potential according to the following formula (Krumperman 1983):

$$MAR{\text{ }}index\, = \,\frac{{Number~of~antibiotics~that~isolate~is~resistant~}}{{Total~number~of~antibiotics~tested}}$$

#### Antibacterial activity against pathogens

The antibacterial activity of *L. plantarum* BG24 and reference strains was determined by using agar well diffusion method by Argyri et al. (2013) with some modifications. All strains were tested in triplicates against *S. aureus* ATCC 6538P, *L. monocytogenes* Scott A, *E. coli* O157:H7 ATCC 35150, *S.* Typhimurium NRRL-B4420. 24 h-old freshly MRS culture were centrifuged (3000*×g*, 10 min, 4 °C) and supernatants were filter-sterilized (0.45 μm). The cell-free culture supernatants (CFC) of the LAB strains were used for inhibitory activity tests.

An overnight culture of pathogens grown in TSB media at 37 °C was used to prepare the culture suspension in sterile Phosphate Buffer Saline (PBS, pH 7.4). The culture suspensions were adjusted to 0.5 McFarland standards (≅1.5 × 10^8^ cfu/mL) and diluted 10-fold in sterile PBS. One hundred microliter of suspension was inoculated on the surface of Nutrient Agar (NA; pH 7.0 ± 0.2, Merck, Germany) by spread plating method and the petri dishes were keep for 15 min to dry in the biosafety cabinet (Esco Class II, Singapore). Then, 100 µL of CFC was transferred in hole (8 mm diameter) drilled into the agar. The plates were incubated at 37 °C for 24 h, and the antibacterial activity was recorded as growth-free inhibition zones (diameter) around the well.

#### Adherence to Caco-2 cells

The adherence ability of *L. plantarum* BG24 and reference strains to human intestinal epithelial Caco-2 cells was examined by using the method by Argyri et al. (2013) with some modification. 24-well plates were used to seed 2 × 10^5^ Caco-2 cells with Dulbeco’s modified Eagle’s medium (DMEM; 2 mM glutamine, 1% (w/v) nonessential amino acids, 20% (v/v) fetal bovine serum) supplemented with 100 U/mL penicillin/streptomycin. The fresh medium was added in every 24 h and monolayers were maintained for 7 days in 5% CO_2_ at 37 °C. Two hours before co-incubation the medium in each well was replaced with pre-warmed fresh medium without antibiotics.

*L. plantarum* BG24 strain was grown in MRS broth at 37 °C for 24 h and the fresh culture was washed twice with sterile PBS (centrifugation at 7,000*×g*, 5 min, 4 °C). Next, approximately 10^7^ cfu were added to each well and the plate was incubated at 37 °C for 4 h. After co-incubation, the cells were washed twice with PBS and 2 mL Triton X-100 (1%, v/v) was added to lyse. After 5 min of incubation at 37 °C, cell lysates were serially diluted and plated on MRS agar by spread plating method. The adherence was calculated by the ratio of the number of bacterial cells that remained attached to the total number of bacterial cells added initially to each well. The experiment was repeated 6 times.

#### Enzyme profile

The enzyme activities of *L. plantarum* BG24 and reference strains were evaluated by API ZYM kit (BioMerieux, France). Briefly, 65 µL of bacterial samples were inoculated in each cupule and incubated for 4 h at 37 °C. Then, a drop of ZYM A and ZYM B reagents was added to each cupule, and the level of enzyme activity was scored as 0 (no activity) to 5 based on color change.

#### Probiotic potential calculation

The probiotic potential of BG24 strain is based upon cumulative probiotic score according to the method by Tambekar and Bhutada (2010) with some modifications. Each property of LAB strains was scored as Table 1. Cumulative probiotic potential is the sum of the score of acid and bile tolerance, antibacterial activity against four pathogens, adherence to Caco-2 and MAR scores. Probiotic potential was calculated as observed score divided by maximum score into hundred by using following formula:

$${\text{Probiotic potential}}\, = \,\frac{{{\text{Observed~score}}}}{{{\text{Maximum~score}}}} \times 100$$

**Table 1:** The indication and scores of probiotic properties used in calculation of probiotic potential of *L. plantarum* BG24 and reference strains

Probiotic properties	Indication	Score
Tolerance to pH	pH 2.0 pH 3.5 pH 5.0	1 1 1
Tolerance to 6.5% NaCl	Resistant	1
	Sensitive	0
Antibacterial activity – *E. coli* O157:H7	−	0
	+	1
	++	2
	+++	3
Antibacterial activity – *S.* Typhimurium	−	0
	+	1
	++	2
	+++	3
Antibacterial activity – *S. aureus*	−	0
	+	1
	++	2
	+++	3
Antibacterial activity – *L. monocytogenes*	−	0
	+	1
	++	2
	+++	3
Adherence to Caco-2 (%)	< 10	0
	≥ 10, < 20	1
	≥ 20	2
MAR index	< 0.2	0
	≥ 0.2	1
Maximum score		19

*MAR Index: Multiple Antimicrobial Resistance Index

### The optimization of growth conditions for *L. plantarum* BG24

#### Inoculum preparation and fermentation conditions

The culture from glycerol stock was subcultured at 37 °C for 24 h and stored at 4 °C. Microorganism was grown in MRS broth at 37 °C for 18–20 h, the time required for the *L. plantarum* BG24 to reach the exponential growth phase. Effect of different inoculum ratios (1, 2, 3, 4, 5; v/v), initial pH values (5.5, 5.7, 6.0, 6.5, 7.0), shaking speeds (0, 100 rpm, 200 rpm), glucose (Merck, Germany) supplementation (5, 10, 15, 20; g/L) and yeast extract (Merck, Germany) supplementation (5, 10; g/L) was examined to achieve maximum growth of *L. plantarum* BG24. Experiments were carried out in 250 mL flasks containing 50 mL of fermentation medium. Fermentation was started with 1% (v/v) inoculum ratio at pH 5.7 under static growth conditions with MRS broth and without any additional nutrients. Samples were collected for OD_600_ measurements at 18th h of fermentation, where maximum OD_600_ values were obtained. One factor at a time method was used for optimization and the condition that yielded the highest bacterial growth (OD_600_) was used for further experiments.

#### Calculation of kinetic parameters for *L. plantarum* BG24 growth

Cell growth was investigated through measurements of optical density at 600 nm (OD_600_), viable cell counts, and biomass quantification. Samples were taken at each 2 h during fermentation for 24 h. Viable cell counts were enumerated using pour plating method (Choi et al. 2021). The fermentation broth was diluted ten-fold, 1 mL of inoculum was inoculated to MRS agar by pour plating technique and then incubated for 48 h at 37 °C. Colony counts were performed and the number of *L. plantarum* BG24 was calculated as log cfu/mL.

Biomass was measured using the method of Üçok and Sert (2020) with some modifications. The fermented broth was centrifuged at 6,000*×g* for 15 min at 4 °C (Daihan Scientific CF-10, Korea), and supernatant was removed. Cell pellets were washed twice with sterile deionized water and dried in a drying oven (Memmert UN55, Memmert GmbH, Germany) at 60 °C for 24 h. Dry cells were weighed by a precision balance (Precisa XB 220 A, Switzerland). Growth kinetics of *L. plantarum* BG24 were calculated using the mathematical expressions below, where µ: Specific cell growth rate (h^−1^), t_d_: Doubling time (h), t: time (h), X: Biomass (g/L), X_0_: Initial biomass (g/L), X_max_: Maximum biomass (g/L), dX/dt: Cell growth rate (gL^−1^ h^−1^)

1 $$\mu =\frac{1}{X}\frac{dX}{dt} \mu =(lnX\text{1}-X\text{0})/t$$

2 $$Productivity= (Xmax -X0) /(tmax-t0)$$

3 $$X=2X\text{0}\text{ } \mu =ln 2/ td td =0.693/ \mu$$

The value of specific growth rate was calculated from the slope of the graph; the natural logarithm of *L. plantarum* BG24 growth plotted against time (Göksungur 2011). Productivity was calculated as the biomass concentration produced per unit time per liter of culture volume. Growth can also be expressed as dry mass concentration (g L^−1^), calculated according to the equation obtained from a calibration curve using *L. plantarum* BG24 dry cells and optical density. From the biomass- OD graph, a correlation equation was obtained as shown below,

4 $$y\, = \,0.3519{\text{x}}\, - \,0.1306\,(R^{2} \, = \,0.9903)$$

Optical density of the fermentation media was measured by using spectrophotometer (Thermo Scientific, Genesys 10 S UV–Vis, USA) at 600 nm. Dilutions and blanks were prepared with sterile MRS broth without inoculation. Each assay was performed in triplicate.

#### Production of *L. plantarum* BG24 in stirred tank bioreactor

The experiments for optimizing the growth of *L. plantarum* BG24 were carried out in a 5-liters stirred tank bioreactor (Sartorius Biostat Aplus, Germany) with 2-liters working volume. The bioreactor is made up with a glass vessel with 4 equally spaced vertical baffles, and 6 cm diameter of dual Rushon-style impellers performed the agitation. The fermentation medium and the vessel were sterilized for 30 min at 121 °C, the pH of medium was adjusted to 6.50 and not further controlled during the process. The fermentation temperature was maintained at 37 °C. Oxygen tension was quantified by determining the percentage of dissolved oxygen (DO%) relative to air saturation, employing an oxygen electrode (InPro 6830; Mettler Toledo, Switzerland). Aeration rate (0–1 vvm), agitation speed (0–500 rpm), inoculation rate (1–10% v/v) and fermentation time (2–24 h) were used as the optimization parameters. The experimental design was planned by using Design Expert 7.0 (Table 2).

**Table 2:** Analysis of variance (ANOVA) test for Box-Behnken factorial design

Source	Sum of squares	df	Mean square	F-value	*p*-value Prob > F	
Model	26.75	14	1.91	122.99	< 0.0001	Significant
A-Aeration rate	1.30	1	1.30	83.68	< 0.0001	
B-Agitation speed	1.40	1	1.40	90.15	< 0.0001	
C-Inoculation rate	7.500E-003	1	7.500E–003	0.48	0.5004	
D-Time	12.71	1	12.71	818.00	< 0.0001	
AB	0.36	1	0.36	23.17	0.0004	
AC	0.051	1	0.051	3.26	0.0962	
AD	0.12	1	0.12	7.88	0.0158	
BC	0.39	1	0.39	25.14	0.0003	
BD	5.625E-003	1	5.625E–003	0.36	0.5586	
CD	0.040	1	0.040	2.57	0.1346	
A^2^	2.03	1	2.03	130.53	< 0.0001	
B^2^	2.07	1	2.07	133.19	< 0.0001	
C^2^	0.56	1	0.56	35.79	< 0.0001	
D^2^	2.87	1	2.87	184.59	< 0.0001	
Residual	0.19	12	0.016			
*Lack of Fit*	0.18	10	0.018	7.26	0.1271	Not significant
*Pure Error*	5.000E-003	2	2.500E–003			
Cor Total	26.94	26				

#### Determination of the biomass, optical density, and viable cell counts

*L. plantarum* BG24 was activated two times in sterile MRS broth (pH 5.7 ± 0.2) at 37 °C for 24 and 18 h, respectively. Then, the active *L. plantarum* BG24 cultures were inoculated (10%, v/v) to MRS broth in 250 mL Erlenmeyer flask and incubated at 37 °C for 18 h in order to prepare fresh working culture. Finally, the fresh working culture was used to inoculate 2 L of MRS broth in the 5-liters stirred tank bioreactor.

The average values of each independent variable were used for carrying out a trial production. 110 mL (5.5%, v/v) of the fresh working culture was inoculated in 2 L of sterile MRS broth in the bioreactor. The working parameters were adjusted as follows: Shaking speed: 250 rpm, ventilation: 0.5 vvm, and temperature: 37 °C. Samples were taken from the bioreactor at 2-h intervals for 24 h. The biomass and optical density analysis were performed in every 2 h while the total viable count analysis was performed every 4 h.

The biomass of *L. plantarum* BG24 was determined by using dry weight measurement. Briefly, 2 mL of samples taken from the bioreactor was centrifuged at 7500 rpm for 20 min. The supernatant was removed, and cell pellets were dried at 60 °C for 24 h until reaching a constant weight, then weighed on the precision scale to obtain dry biomass weight. Optical density was measured by using a spectrophotometer at 600 nm. Dilutions and blanks were prepared with sterile MRS broth without inoculation. The viable cell counts of *L. plantarum* BG24 were applied by pour plate method as explained in previous sections.

#### Optimization of the biomass by Box-Behnken design

The effects of independent variables were determined by employing a Box-Behnken design on the response and factor interactions (Table 2). As mentioned in previous studies (Mahdinia et al. 2019; Silva-Santisteban et al. 2005; Zhong 2010), aeration rate, stirring rate (agitation speed), inoculation rate, and fermentation time are the four major parameters affecting the fermentation process, these factors are selected to optimize for *L. plantarum* BG24 fermentation. The process parameters affecting the biomass (g/L) were investigated and validated with a three-level and four independent variables design. The independent variables which are aeration rate (0 [no aeration], 0.50 and 1.00; vvm), agitation speed (0 [no agitation], 250 and 500 rpm), inoculation rate (1%, 5.5% and 10%; v/v) and time (2, 13 and 24, h) were studied at three levels which were − 1, 0 and + 1, corresponding to the low, medium, and high values, respectively.

#### Statistical analysis

Significant differences between the mean values were determined using Tukey’s multiple comparison tests using SPSS 25.0 (SPSS Inc., New York, USA). The mean values for all the factors given in the x-axis that are not followed by the same letter (a–d) are significantly different (p < 0.05). The results were given as mean ± standard deviation.

## Results

### Probiotic potential of *L. plantarum* BG24

In the present study, *L. plantarum* BG24 and reference strains (Visby and LA5) showed a tolerance to all bile salt concentrations (0.5, 1.0, 1.5 and 2.0%, w/v) as showed in Table 3. On the other hand, only *L. plantarum* BG24 had a tolerance to all pH values (2.0, 3.5 and 5.0). Reference strain *L. plantarum* Visby did not survive at pH 2.0 while *L. acidophilus* LA5 survived only at pH 5.0 value. All LAB strains (BG24, Visby and LA5) were able to survive at the temperature values of 10 °C, 25 and 45 °C (Table 3). *L. plantarum* BG24 and *L. plantarum* Visby were able to tolerate both 4.0 and 6.5% (w/v) of NaCl concentrations however *L. acidophilus* LA5 did not survive at the concentration of 6.5% (w/v) of NaCl (Table 3). *L. plantarum* BG24 and both reference strains were grown in fermentation medium supplemented with 5 g/L of lactose and it was observed that color change from red to yellow based on the production of lactic acid from lactose.

**Table 3:** Growth of *L. plantarum* BG24 and reference strains under different conditions

LAB strain	pH value			Bile salt concentration (%, w/v)				Temperature (°C)			NaCl (%, w/v)		Lactose utilization
	2.0	3.5	5.0	0.5	1.0	1.5	2.0	10	25	45	4.0	6.5	
BG24	+	+	+	+	+	+	+	**+**	**+**	**+**	**+**	**+**	**+**
Visby	−	+	+	+	+	+	+	**+**	**+**	**+**	**+**	**+**	**+**
LA5	−	−	+	+	+	+	+	**+**	**+**	**+**	**+**	**−**	**+**

“+”: Growth, “−”: No growth

*L. plantarum* BG24 and reference strains were tested for their susceptibility to nineteen different antibiotics and the results were given in Table 4. All strains were found to be resistant towards three antibiotics that inhibit the protein synthesis (Amikacin, Kanamycin and Tobramycin) and two antibiotics that inhibit the nucleic acid synthesis (Ciprofloxacin and Nalidixic acid). *L. acidophilus* LA5 strain was susceptible to all antibiotics which are inhibitors of cell wall synthesis while *L. plantarum* BG24 and *L. plantarum* Visby strains showed resistance to Vancomycin and Oxacillin. On the other hand, the MAR indexes of *L. plantarum* BG24, *L. plantarum* Visby and *L. acidophilus* LA5 were calculated as 0.421, 0.368 and 0.316, respectively (Table 4).

**Table 4:** Antibiotic susceptibility and MAR index of *L. plantarum* BG24 and reference strains

	Inhibitors of cell wall synthesis							Inhibitors of protein synthesis										Inhibitors of nucleic acid synthesis		
**LAB** **strain**	Penicillin	Ampicillin	Cefamandole	Vancomycin	Piperacillin	Amoxicillin	Oxacillin	Doxicycline	Gentamicin	Kanamycin	Lincomycin	Tobramycin	Amikacin	Tetracyclin	Erithromycin	Rifampin	Chloramphenicol	Ciprofloxacin	Nalidixic acid	**MAR Index**
**BG24**	S	S	S	R	S	S	R	I	R	R	S	R	R	S	S	S	S	R	R	8/19 = 0.421
**Visby**	S	S	S	R	S	S	R	S	I	R	R	R	R	I	I	S	S	R	R	7/19 = 0.368
**LA5**	S	S	S	S	S	S	S	S	R	R	S	R	R	S	S	S	S	R	R	6/19 = 0.316

“R”: resistant, “S”: Sensitive, “I”: Intermediately susceptible

The antibacterial activity of *L. plantarum* BG24 and reference strains were determined by agar well diffusion method against two Gram-positive and two Gram-negative pathogens and the results were given in Table 5. *L. plantarum* BG24 showed the highest inhibitory effect against *E. coli* O157:H7 however the inhibitory effect of *L. acidophilus* LA5 was recorded as negative. All LAB strains were able to inhibit the growth of *S.* Typhimurium, and *L. plantarum* Visby showed the biggest zone of inhibition (17 mm, strong effect) following by *L. plantarum* BG24 (14 mm, weak effect) and *L. acidophilus* LA5 (13 mm, weak effect). The zone of inhibition diameter was found to be equal (16 mm, weak effect) on *S. aureus* for *L. plantarum* BG24 and *L. plantarum* Visby. However, no inhibitory effect was observed for *L. acidophilus* LA5 against *S. aureus.* In addition, *L. monocytogenes* was the most sensitive pathogen against LAB strains tested in the study. All LAB strains showed inhibitory effect on the growth of *L. monocytogenes* and the zone of inhibition of BG24, Visby and LA5 strains were 26 mm (very strong effect), 27 mm (very strong effect) and 19 mm (strong effect), respectively.

**Table 5:** Antimicrobial activity of *L. plantarum* BG24 and reference strains against different Gram-positive and Gram-negative pathogens by agar well diffusion method

LAB strain	*E. coli* O157:H7	*S.* Typhimurium	*S. aureus*	*L. monocytogenes*
BG24	16 mm (+)	14 mm (+)	16 mm (+)	26 mm (+++)
Visby	14 mm (+)	17 mm (++)	16 mm (+)	27 mm (+++)
LA5	11 mm (−)	13 mm (+)	8 mm (−)	19 mm (++)

*Zone of inhibition assessment (Jomehzadeh et al. 2020): 11 mm or less (negative, −); 12–16 mm (weak effect, +); 17–22 mm (strong effect, ++); 23 mm and larger (very strong effect, +++). *Results include 8 mm well diameter

The adhesion ability of *L. plantarum* BG24 was determined as 37.51% to Caco-2 cells while it was found as 14.61% and 12.41% for *L. plantarum* Visby and *L. acidophilus* LA5, respectively. The API ZYM kit was used to confirm the degree of 19 enzymes production for *L. plantarum* BG24, *L. plantarum* Visby and *L. acidophilus* LA5 (Table 6). *L. plantarum* BG24 did not produce Alkaline phosphatase, Lipase (C14), Trypsin, α-chymotrypsin, α-galactosidase, β-glucuronidase, α-mannosidase and α-fucosidase. On the other hand, Leucine arylamidase, β-glucosidase, Valine arylamidase, β-galactosidase and N-acetyl-β-glucosaminidase were highly produced by *L. plantarum* BG24.

**Table 6:** Enzymatic activity profile of *L. plantarum* BG24 and reference strains

No	Enzym	Score			
		BG24	Visby	LA5	
1	Control	0	0	0	
2	Alkaline phosphatase	0	0	0	
3	Esterase (C4)	1	0	1	
4	Esterase Lipase (C8)	1	0	1	
5	Lipase (C14)	0	0	0	
6	Leucine arylamidase	5	5	5	
7	Valine arylamidase	4	4	1	
8	Cystine arylamidase	2	2	1	
9	Trypsin	0	0	0	
10	α-chymotrypsin	0	0	0	
11	Acid phosphatase	1	1	2	
12	Naphtol-AS-BI-phosphohydrolase	2	1	1	
13	α-galactosidase	0	0	0	
14	β-galactosidase	4	5	1	
15	β-glucuronidase	0	0	0	
16	α-glucosidase	1	1	1	
17	β-glucosidase	5	5	1	
18	N-acetyl-β-glucosaminidase	4	2	0	
19	α-mannosidase	0	0	0	
20	α-fucosidase	0	0	0	

**Fig. 1:**
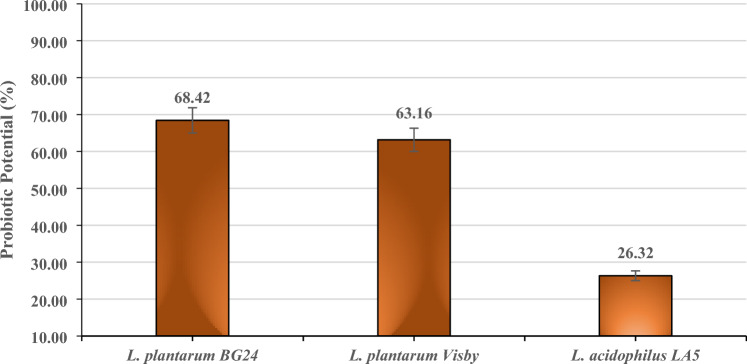
The probiotic potential of *L. plantarum* BG24 and reference strains

The probiotic potential of *L. plantarum* BG24 isolated from boza was calculated according to the cumulative probiotic scores obtained by various analyzes and compared with reference LAB strains (Fig. 1). The highest probiotic potential was found as 68.42% for *L. plantarum* BG24 following by *L. plantarum* Visby (63.16%) and *L. acidophilus* LA5 (26.32%).

### The optimization of growth conditions for *L. plantarum* BG24

**Fig. 2:**
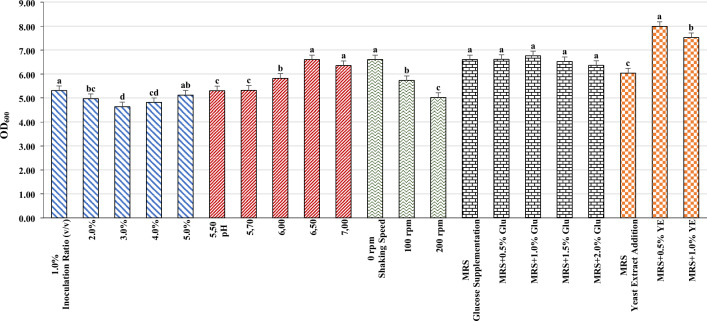
Effect of different parameters (inoculation ratio, pH, shaking speed, glucose supplementation and yeast extract addition) on *L. plantarum *BG24 growth after 18 h incubation. Different letters (a–d) on data bars indicate a significant difference (p < 0.05) between mean values. Statistical analyzes were applied separately for each parameter

The growth of *L. plantarum* BG24 in MRS broth was examined and the effect of different factors (inoculum ratio, initial pH) and different supplements (glucose, yeast extract) on microbial growth was determined. First, different inoculation ratios (1–5%, v/v) were investigated to maximize the growth *of L. plantarum* BG24. The highest OD_600_ value of 5.31 ± 0.09 was obtained at the inoculation rate of 1% (v/v) after 18 h of fermentation (Fig. 2). Although higher OD_600_ values were obtained in the exponential phase when high inoculation rates (2, 3, 4, 5%; v/v) were used (data not shown), the inoculation rate of 1% (v/v) yielded the highest OD_600_ value at the end of the exponential phase.

The effect of the initial pH values (5.5, 5.7, 6.0, 6.5, 7.0) on *L. plantarum* BG24 growth was studied. As seen in Fig. 2, the highest OD_600_ value of 6.60 ± 0.06 was observed at pH 6.5 while similar OD_600_ value of 6.35 ± 0.04 was obtained at a pH value of 7.0. Below the 6.5 pH values, microbial growth was reduced. This result indicated that neutral pH values are favorable for *L. plantarum* BG24 growth. Different shaking speeds were employed to examine the oxygen demand of *L. plantarum* BG24. Inoculated flasks were incubated under static condition without shaking and aerobic condition at 100 and 200 rpm. The fermentation was carried out at 37 °C in 250 mL conical flasks containing 50 mL of fermentation medium (pH 6.5). The flasks were inoculated with 1% (v/v) of *L. plantarum* BG24. Shake flask experiments were applied in a rotary shaker incubator (IKA KS 260, Türkiye) operated at 100 or 200 rpm. Increased shaking speeds resulted in a decrease in growth of *L. plantarum* BG24 (Fig. 2). Maximum growth was obtained with an OD_600_ value of 6.60 ± 0.24 OD_600_ with static culture when no shaking was employed.

Fermentation medium (MRS broth) was supplemented with 5, 10, 15, 20 g/L and glucose to determine the effect of glucose supplementation on microbial growth of *L. plantarum* BG24. Fermentation was performed under static culture conditions at 37 °C and pH 6.5. As seen in Fig. 2, no significant differences (p > 0.05) were observed in OD_600_ values of *L. plantarum* BG24 grown in MRS medium containing different concentrations of glucose. These results suggest that supplementing the fermentation medium with glucose did not enhance microbial growth of *L. plantarum* BG24. Although the differences were not statistically significant (p > 0.05), the highest OD_600_ value (6.76 ± 0.11 OD_600_) was obtained when 10 g/L glucose supplementation was used. Maximum OD_600_ values were obtained at the 20th h of fermentation when glucose was supplemented to the fermentation medium while this value was 18 h when no glucose was used in the fermentation medium (data not shown). To investigate the effect of yeast extract concentration on microbial growth of *L. plantarum*, 5 and 10 g/L of yeast extract was added to the fermentation medium (MRS broth) and fermentation was carried out under static culture conditions at 37 °C and pH 6.5 until the stationary phase. As seen in Fig. 2, yeast extract supplementation had a significant effect on *L. plantarum* BG24 growth. The highest OD_600_ value of 7.99 ± 0.15 was obtained when the fermentation medium was supplemented with 5 g/L yeast extract. At 10 g/L yeast extract concentration, OD_600_ value of 7.52 ± 0.08 was obtained which was still higher than the OD_600_ value of the fermentation medium with no yeast extract supplementation. The results showed that yeast extract promotes the growth of *L. plantarum* BG24 when it was used as a nitrogen source.

**Fig. 3:**
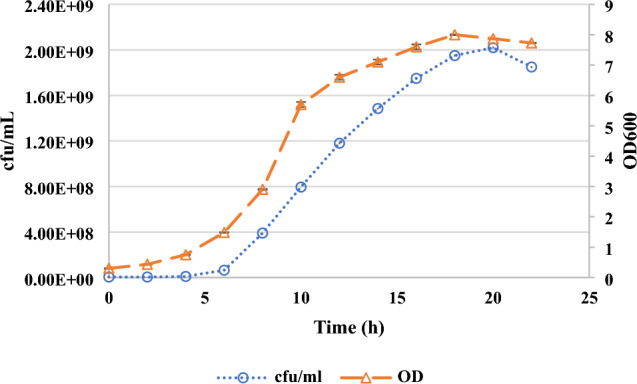
Growth of *L. plantarum* BG24 in MRS broth supplemented with 5 g/L yeast extract (37 °C, initial pH 6.5, 1% [v/v] inoculation ratio, static conditions). The standard deviation of each experimental point ranged from 1.2 to 4.5

In order to determine the growth kinetics of *L. plantarum* BG24, fermentation was carried out in MRS broth supplemented with 5 g/L yeast extract at 37 °C with an inoculation ratio of 1% (v/v). The fermentation was carried out under static culture conditions with an initial pH of 6.5. *L. plantarum* BG24 growth was observed during the fermentation and kinetic parameters were calculated. Microbial count and OD_600_ curves were given in Fig. 3. *L. plantarum* BG24 growth was characterized by a lag phase of about 4 h followed by an exponential phase until 18–20 h of fermentation. Bacterial count of 2.02 × 10^9^ cfu/mL (9.31 log cfu/mL) was obtained after 20 h of fermentation when *L. plantarum* BG24 reached the stationary phase.

**Fig. 4:**
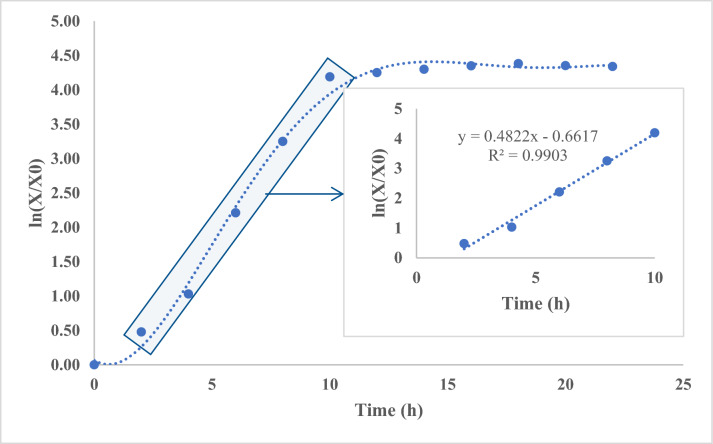
Natural logarithm of *L. plantarum* BG24 grown in MRS broth and calculation of specific growth rate. The standard deviation of each experimental point ranged from 1.8 to 4.7

The increase in cell number during microbial growth showed a parallel trend to the increase in dry biomass concentration (data not shown). The highest OD_600_ value and biomass concentration were obtained as 8 OD_600_ and 2.6 g/L, respectively on the 18th h of the fermentation (Fig. 4). Specific growth rate (µ) was found as 0.4822 h^−1^ (Eq. ) by using the slope of the graph, which plotted the natural logarithm of bacterial growth against time (Fig. 4). Using Eq. 2, the productivity of *L. plantarum* BG24 was found as 0.17 gL^−1^ h^−1^and by using Eq. 3, doubling time for *L. plantarum* BG24 was found as 1.43 h. In the optimized growth conditions, specific growth rate (from 0.416 to 0.482 h^−1^) and productivity values (from 0.14 to 0.17 gL^−1^ h^−1^) were increased, and normally doubling time (from 1.67 to 1.43 h) was decreased for *L. plantarum* BG24 .

### Optimization of the growth of *L. plantarum* BG24 in stirred tank bioreactor by Box Behnken design

Our preliminary experiments in stirred tank bioreactor showed that the inoculation rate (v/v), aeration rate (vvm), agitation speed (rpm) and incubation time (h) influenced the biomass production (g/L) of *L. plantarum* BG24 in stirred tank bioreactor. The levels of these factors (Table 7) used in the optimization studies by RSM were determined by previous experiments (data not shown).

**Table 7:** Experimental design for the optimization of the production of *L. plantarum* BG24; Independent variables (Aeration rate, Agitation speed, Inoculation rate, Time) and dependent variable (Biomass)

			Factor 1	Factor 2	Factor 3	Factor 4	Response
Std	Run	Block	A: Aeration (vvm)	B: Agitation (rpm)	C: Inoculation rate (%)	D: Time (h)	Biomass (g/L)
6	1	Block 1	0.50	250.00	10.00	2.00	1.1
26	2	Block 1	0.50	250.00	5.50	13.00	2.3
3	3	Block 1	0.00	500.00	5.50	13.00	3.8
9	4	Block 1	0.00	250.00	5.50	2.00	1.3
18	5	Block 1	1.00	250.00	1.00	13.00	3
5	6	Block 1	0.50	250.00	1.00	2.00	0.8
2	7	Block 1	1.00	0.00	5.50	13.00	3.9
10	8	Block 1	1.00	250.00	5.50	2.00	0.9
15	9	Block 1	0.50	0.00	10.00	13.00	3.9
12	10	Block 1	1.00	250.00	5.50	24.00	2.6
11	11	Block 1	0.00	250.00	5.50	24.00	3.7
20	12	Block 1	1.00	250.00	10.00	13.00	2.75
22	13	Block 1	0.50	500.00	5.50	2.00	0.75
8	14	Block 1	0.50	250.00	10.00	24.00	2.8
7	15	Block 1	0.50	250.00	1.00	24.00	2.9
14	16	Block 1	0.50	500.00	1.00	13.00	3.1
19	17	Block 1	0.00	250.00	10.00	13.00	3.5
23	18	Block 1	0.50	0.00	5.50	24.00	3.5
1	19	Block 1	0.00	0.00	5.50	13.00	4
4	20	Block 1	1.00	500.00	5.50	13.00	2.5
27	21	Block 1	0.50	250.00	5.50	13.00	2.2
21	22	Block 1	0.50	0.00	5.50	2.00	1.2
16	23	Block 1	0.50	500.00	10.00	13.00	2.55
25	24	Block 1	0.50	250.00	5.50	13.00	2.25
24	25	Block 1	0.50	500.00	5.50	24.00	2.9
17	26	Block 1	0.00	250.00	1.00	13.00	3.3
13	27	Block 1	0.50	0.00	1.00	13.00	3.2

Analysis of variance (ANOVA) test for biomass production (g/L) of *L. plantarum* BG24 was given in Table 2. The analysis gives the value of the model and determines the requirement of a more complex model with a better fit. R^2^ value was found as 0.9931 indicating that the model as fitted, and F-value was found as 122.99 which implied the model to be significant. Model terms having values of Prob > F are considered highly significant. In addition, if the F-test for lack of fit is significant, a more complicated model is needed to fit the data.

As shown in Table 2, the lack of fit was insignificant. Twenty-seven experiments were carried out from the design and by applying multiple regression analysis on the experimental data, the following second- order polynomial equation was found to explain the biomass production of *L. plantarum* BG24:

5 $${\text{Biomass }} = {\text{ }} + {\text{2}}.{\text{25 }}{-}{\text{ }}0.{\text{33A }}{-}{\text{ }}0.{\text{34B }} + {\text{ }}0.0{\text{25C }} + {\text{ 1}}.0{\text{3D }}{-}{\text{ }}0.{\text{3}}0{\text{AB }}{-}{\text{ }}0.{\text{11AC }}{-}{\text{ }}0.{\text{17AD }}{-}{\text{ }}0.{\text{31BC }}{-}{\text{ }}0.0{\text{38BD }}{-}{\text{ }}0.{\text{1}}0{\text{CD }} + {\text{ }}0.{\text{62A}}^{{\text{2}}} + {\text{ }}0.{\text{62B}}^{{\text{2}}} + {\text{ }}0.{\text{32C}}^{{\text{2}}} - {\text{ }}0.{\text{73D}}^{{\text{2}}}$$

where A, B, C and D are the actual levels of factors shown in Table 7.

**Fig. 5:**
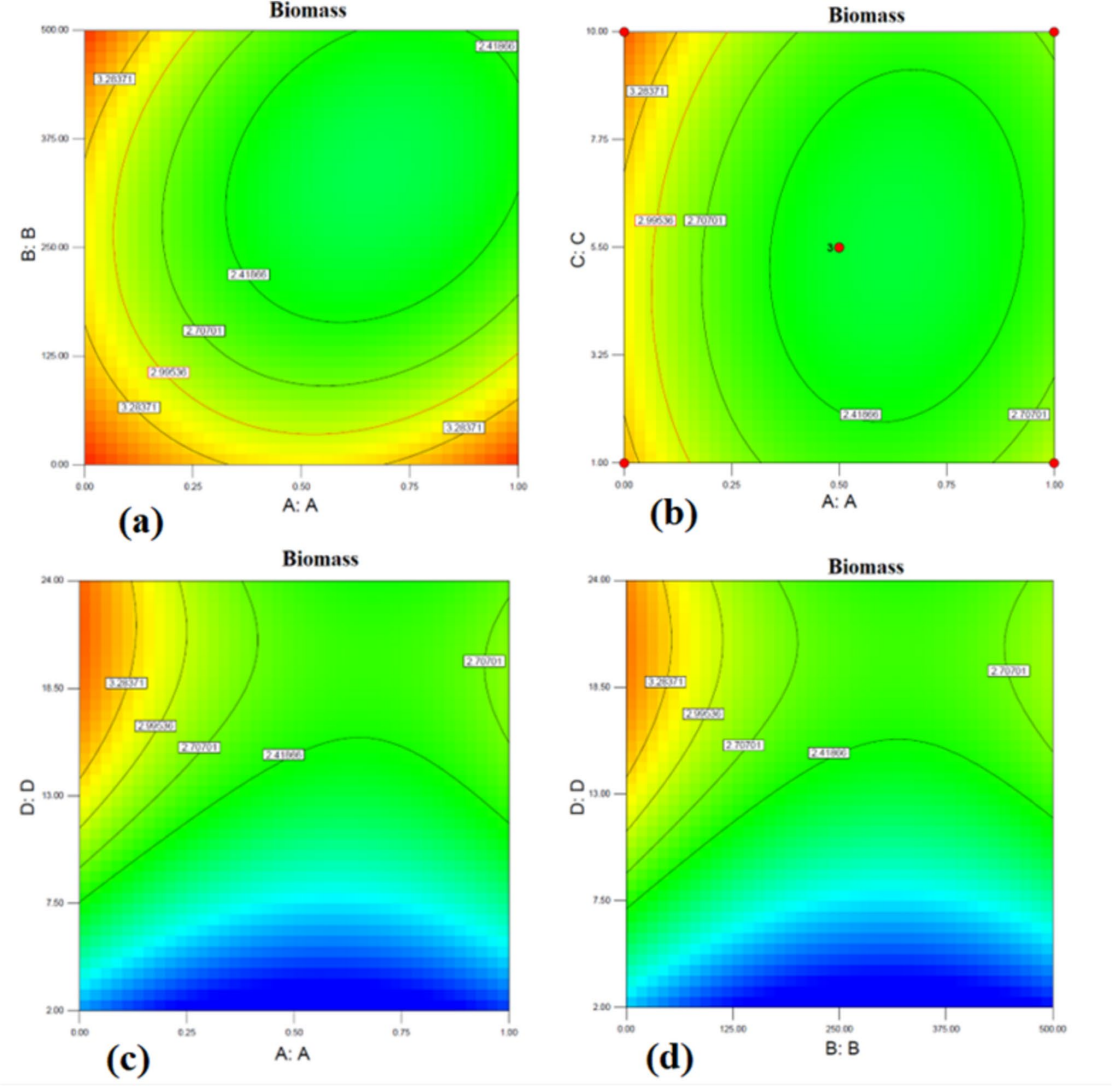
Contour plots of interactions between Aeration rate (**A**), Agitation speed (**B**), Inoculation rate (**C**) and Time (**D**) for the biomass production of *L. plantarum* BG24. (**a**):Aeration rate: Agitation speed, (**b**): Aeration rate: Inoculation rate, **(c):** Aeration rate: Time, (**d**): Agitation speed: Time

Figure 5 shows the response surface plots of biomass concentration for each pair of factors whereas the other factors were kept constant at their middle levels. As shown in Fig. 5a, for the same levels of aeration rate and agitation speed, biomass concentration decreased from the middle to high aeration rate or agitation speed levels. Figure 5b shows that biomass concentration increased with the increase in aeration rate while inoculation rate has less effect on biomass concentration. Figure 5c and d also showed similar trends showing that biomass concentration increased and reached a maximum at the middle level of variables; namely agitation speed and fermentation time (Fig. 5c) and aeration rate –fermentation time (Fig. 5d). Further increases in the above factors resulted in a decrease in biomass concentration.

In order to find the maximum biomass concentration corresponding to the optimum levels of aeration rate, agitation speed, inoculation rate and fermentation time, a second order polynomial model was used to calculate the values of these process variables. The fitting of the experimental data to Eq. 5 allowed the determination of the levels of aeration rate (A = 0.05 vvm), agitation speed (B = 478.75 rpm), inoculation rate (C = 2.98%), and time (D = 17.48 h) giving a maximum biomass concentration of 4.0775 g/L using Design Expert 7.0. The above data optimizes *L. plantarum* BG24 production in a laboratory scale stirred tank bioreactor. The final fermentation (validation experiment) was performed using MRS medium in the 5 L stirred tank bioreactor with the optimized levels of the process parameters (Agitation speed: 479 rpm, Aeration: 0.05 vvm, Inoculation rate(v/v): 3%, Time: 18 h). According to the results of the validation experiment, the OD_600_ value, biomass production and viable cell counts of *L. plantarum* BG24 were found as 9.6 OD_600_, 3.84 g/L and 7.6E + 11 cfu/mL, respectively.

## Discussions

Tolerance to high acidity and high concentration of bile components are the two major conditions for a probiotic strain since a probiotic organism ingested with food must survive the transit through the upper gastrointestinal tract, cross all barriers and remain in a viable physiological state to benefit the host (García-Hernández et al. 2016; Padmavathi et al. 2018). The bile salt tolerance is a prerequisite for colonization and metabolic activity of bacteria in the small intestine so that LAB strains could reach the small intestine and colon for balancing the intestinal microflora (Foley et al. 2021; Tambekar and Bhutada 2010). Todorov et al. (2012) indicated that a probiotic bacteria must pass through the stomach (pH 1.5–2.0) before reaching the intestinal tract. In this study, potential probiotic *L. plantarum* BG24 showed the highest tolerance to the different pH values compared the other reference strains.

The ability of LAB strains to survive in adequate amount at low or high temperature is an important characteristic for their use in food industry (Kazancıgil et al. 2019). The LAB strains which had NaCl tolerance could withstand the adverse effects of high osmotic pressure in the gastrointestinal tract (Xu et al. 2019). Forhada et al. (2016) isolated various probiotic lactic acid bacteria from buffalo milk and reported that while all the isolates were able to grow at 1–6% (w/v) of NaCl concentrations, they could not survive at 7–10% (w/v) of NaCl. Lactose utilization of LAB is one of the most important properties for lactose intolerant people who cannot metabolize lactose because of the lack of the β-galactosidase enzyme. Abdominal pain, cramping or diarrhea arise are some major symptoms for lactose intolerant people who consumed milk or lactose containing products (Sharp et al. 2021). Addition of certain starter cultures like *L. plantarum* BG24 to milk products might be beneficial for the lactose intolerant people to consume those products without any associated symptoms.

A MAR value which is higher than 0.2 was described as indicative of multiple antibiotic resistant bacteria by Krumperman (1983), so all strains tested in the study are characterized as multi-resistant strains (MAR > 0.2). The MAR index introduced in 1983 is widely used to refer multidrug-resistant microorganisms in various environments and any suggestions have not been made regarding the minimum number of antibiotics, yet (Resende et al. 2014). Yadav et al. (2016) isolated four *L. plantarum* strains from traditional fermented beverage Raabadi and reported that the isolates were resistant to Nalidixic acid, Ciprofloxacin, and Vancomycin. Probiotic strains that are resistant to a wide spectrum of antibiotics could be used in controlling the intestinal infections due to the preventive and therapeutic properties of probiotics (El-Naggar 2004). Antibiotic resistant probiotics might be helpful in faster recovery of the patients by establishing rapidly the desirable microbial flora when they were administered at the same time with antibiotics. On the other hand, transmission of the antibiotic resistance genes to pathogenic bacteria is demonstrated as major health concern, so some researchers suggest that probiotics should be sensitive to commonly prescribed antibiotics at the low concentration (Halder et al. 2017; Lee et al. 2012). LAB is expected to be resistant to some antibiotics as a natural or “intrinsic” property. Hummel et al. (2007) stated the mechanism of the intrinsic resistance of LAB to quinolones (Ciprofloxacin) included enzymatic inactivation that preventing the binding of these antibiotics to their specific targets. In addition, the authors attributed the intrinsic resistance to aminoglycosides (Gentamicin) to the absence of electron transport mediated by cytochrome that reducing drug uptake. The EFSA reported bacterial strains carrying intrinsic or chromosomal mutation antibiotic resistance had a minimal to low potential for horizontal resistance spread and generally might be incorporated into foods. It was also considered that bacterial strains carrying antibiotic resistance related to acquired genetic determinants had high potential for horizontal spread and should not be incorporated into foods (EFSA 2012).

Antimicrobial activity is one of the most important criteria for selecting probiotic bacteria. Previous studies approved the bio preservative applications of LAB due to the antimicrobial activities against common foodborne bacteria (Chaillou et al. 2014; dos Santos Leandro et al. 2021; Katiku et al. 2022; Prabhurajeshwar et al. 2017). In the present study, *L. plantarum* BG24 showed antimicrobial properties against four different pathogens. The other important criterion for selecting probiotic bacteria is adhesion to human intestinal epithelial cells. The adhesion property of LAB strains to Caco-2 might provide various beneficial effects such as immune system modulation and competition with the pathogens to colonize the human epithelial cells (Fonseca et al. 2021). The adhesion abilities of *L. paracasei* F7B, *Lactococcus garvieae* S3 and *L. rhamnosus* F9A strains isolated from sourdough and infant feces were found as 9.54, 7.44 and 3.18, respectively to human intestinal HT-29 cells (Kaya et al. 2022). Various LAB strains were isolated from traditionally fermented Turkish beverages, gilaburu and shalgam and ten selected LAB strains showed variable adherence abilities on Caco-2 cells at ranging levels from 37 to 88%. *L. plantarum* XL963 isolated from shalgam showed the highest cell adhesion ability to Caco-2 cells (Akman et al. 2021).

Jeon et al. (2017) declared that LAB should not produce β-glucuronidase since it is a bacterial carcinogenic enzyme which exerts negative effects in the liver. Son et al. (2017) reported β-glucosidase played an important role in bioconversion of ginsenoside, isoflavone and phenolic compound by breaking down of glycosidic bonds. In addition, β-galactosidase enzyme might be beneficial to reduce the lactose intolerance problem by hydrolyzing lactose into glucose and galactose (Saqib et al. 2017). The enzyme profile of *L. plantarum* BG24 was found to be similar to the results of different *L. plantarum* strains in the previous studies (Lee et al. 2006; Nemska et al. 2019).

In the present study, it was clearly showed that *L. plantarum* BG24 isolated from boza was acid tolerant at pH 2.0, resistant to 6.5% of NaCl, antibacterial against different Gram-positive and Gram-negative pathogens, resistant to most of the antibiotics, highly adherence to human intestinal cell Caco-2. These results revealed *L. plantarum* BG24 as a probiotic fulfilled the criteria stated by WHO/FAO (2002).

Characterization of the growth parameters in a commonly accepted media is a crucial step for the commercial application of a probiotic strain. Wardani et al. (2017) investigated the effects of different inoculum rates of 1, 3, 5 and 10% (v/v) on *L. plantarum* growth in skimmed milk medium. Contrary to the results of this study, they observed lower growth on 1% inoculum rate while 10% gave the highest growth after 12 h of incubation. The higher microbial growth obtained with high inoculation ratios at the beginning of the exponential phase might be attributed to a lower adaptation period of those cultures. The pH value of the fermentation medium can have a high impact on the microbial growth because pH influences the physiology of a microorganism significantly by affecting nutrient solubility and uptake, enzyme activity, cell membrane morphology, byproduct formation and oxidative changes (Survase et al. 2007). The optimal pH value of *Lactobacillus plantarum* NCU116 was found as pH 6.5 (Xiong et al. 2011). The highest growth of *L. plantarum* 200,655 was observed at pH 7 and it was stated that low pH conditions (< 4.4) could affect the growth and cause a reduce in the growth rate (Choi et al. 2021). The influence of pH on the growth of *L. plantarum* was investigated using MRS broth as the growth medium and the optimum pH for growth was found as pH 6.0 and pH variations resulted in a decrease in growth rate (Dalcanton et al. 2018; Giraud et al. 1991).

Shaking is crucial in overcoming mass transfer resistances in fermentation systems, and this is directly related with shaker speed. *L. plantarum* is a facultative heterofermentative bacterium whose natural habitats are anaerobic or microaerobic (Krieger-Weber et al. 2020). The results confirm that microaerophilic conditions favor *L. plantarum* BG24 growth. A stationary period was observed in the growth curves of shaked cultures at 12 and 14 h of incubation (data not shown) which might be resulted from the adaptation of the metabolism from microaerophilic to aerobic conditions. Gupta et al. (2010) investigated the growth and kinetics of *L. plantarum* in the fermentation of edible Irish brown seaweeds in order to produce lactic acid. They found that the cell growth increased, but product (lactic acid) formation decreased as the shaking speed was increased from 0 to 100 rpm. They obtained maximum lactic acid concentration of 2.5 g/L under static (0 rpm) conditions.

The effect of initial glucose concentration (1 to 9%; w/v) was investigated on the growth of *L. plantarum* NCU116 in an ammonium and glucose fed-batch system (Xiong et al. 2011). In contrast, they found that the growth of *L. plantarum* increased up to 5% (w/v) glucose concentration in the fed-batch fermentation system and they also stated that higher glucose concentrations might have repressed the growth of *L. plantarum* NCU116. The highest cell growth of a probiotic strain *L. plantarum* was determined in the glucose and lactose supplied MRS medium. Whey permeates and MRS-galacto oligosaccharide were found to highly support the growth and lactic acid production (Georgieva et al. 2009). Yeast extract is the most commonly used nitrogen source in lactic acid fermentation as it provides convenient growth factors for LAB with its high content of nitrogen compounds and vitamin B (Abdullah et al. 2021; Liu et al. 2010). Manzoor et al. (2017) determined the most suitable medium components for the growth of *L. plantarum* AS-14. Contrary to the results of this study, they did not find any significant difference between the 8 g/L yeast extract supplemented media and MRS medium, which originally contains 5 g/L yeast extract. Yoo et al. (2018) optimized the fermentation medium to obtain maximum growth of *L. plantarum* JNU 2116. Their results showed that the presence of yeast extract, moderate glucose, peptone, and magnesium sulfate affected the growth of bacteria. Papizadeh et al. (2020) screened efficient nitrogen sources to yeast extract for optimum growth of the probiotic *L. plantarum* RPR42 in a cane molasses-based medium. They found that a combination of nitrogenous substrates including corn steep liquor, wheat germ extract, cheese whey, and casein hydrolysate can replace yeast extract in medium formulations and promisingly increase the biomass production of *L. plantarum* in a glucose-enriched cane molasses solution.

Growth parameters of *L. plantarum* BG24 were calculated before and after the one-factor-at-a time optimization procedure. Thus, RSM was used to determine the optimum levels of these parameters leading to maximum biomass production. Mecmeche et al. (2017) compared the growth of *L. plantarum* in MRS broth and in protein-rich isolates from tomato seed meal. The biomass concentration, cell concentration, specific growth rate, and maximum productivity values obtained for MRS broth were, 2.024 g/L, 9.45 log cfu/mL, 0.363 h^−1^ and 0.252 gL^−1^ h^−1^, respectively. Those values were considerably higher than the values obtained for tomato seed meal. Üçok and Sert (2020) studied the growth kinetics and biomass characteristics of *L. plantarum* L14 isolated from sourdough in MRS medium. They calculated kinetic parameters as maximum specific growth rate; 0.551 h^−1^, doubling time; 1.26 h^−1^ and productivity; 0.460 gL^−1^ h^−1^. Giraud et al. (1991) found a growth rate of 0.57 h^−1^, a final biomass concentration of 9.5 g/L and productivity of 1.05 g L^−1^ h^−1^ for *L. plantarum* in MRS Broth. Considering the literature data, many factors such as pH, fermentation temperature, fermentation system (batch, continuous or fed-batch) and supplements used affected *L. plantarum* growth although the same synthetic medium (MRS broth) was used as the fermentation medium (Dalcanton et al. 2018; Mecmeche et al. 2017; Siragusa et al. 2014; Üçok and Sert 2020; Zheng et al. 2018; Wardani et al. 2017).

*L. plantarum* was grown in MRS broth under static conditions by Yoo et al. (2018) and the OD_600_ value was found as 1.905 however the OD_600_ value for *L. plantarum* BG24 was higher under both static and stirred conditions in this study. Choi et al. (2021) reported the highest biomass production as 4.304 g/L by *L. plantarum* 200,655 which was grown at pH 7.0. The biomass yield was declared to decrease sequentially above and below this point (between pH 4.0 and pH 9.0). Furthermore, the maximal biomass was observed at 30 °C of fermentation temperature as 4.505 g/L for *L. plantarum* 200,655. The viable cell counts of *L. plantarum* were found in the range of 10^7^–10^10^ cfu/mL after 40–48 h incubation time (Noori et al. 2016; Panda et al. 2008).

The present study investigates the probiotic characteristics of *L. plantarum* BG24 and optimizes the growth conditions of *L. plantarum* BG24. The results showed that the use of *L. plantarum* BG24 in milk and milk products might be beneficial for lactose intolerant patients due to its β-galactosidase activity. The optimal growth parameters for *L. plantarum* BG24 were found as 1% (v/v) of inoculation rate, pH 6.5 and 5 g/L of yeast extract supplementation to standard MRS medium. The growth of *L. plantarum* BG24 was optimized using a stirred tank bioreactor system, resulting in a maximum biomass concentration of 3.84 g/L. This optimal biomass concentration was achieved by setting the process parameters at the following levels: aeration rate of 0.05 vvm, agitation speed of 479 rpm, inoculation rate of 3%, and fermentation time of 18 h. This study employs statistical analysis to identify the optimal levels and interactions of the aforementioned parameters for bacterial growth in a stirred tank bioreactor using *L. plantarum* BG24.The data obtained in the study are expected to guide other researchers looking for new alternative probiotic lactic acid bacterium. Further studies will focus on the pilot scale optimization of the growth of probiotic *L. plantarum* BG24 using 10 and 30 L bioreactors.

## Data Availability

The datasets generated during and/or analysed during the current study are available from the corresponding author on reasonable request.
